# Spatial Modularity of Innate Immune Networks Across Bactrian Camel Tissues

**DOI:** 10.3390/ani15213173

**Published:** 2025-10-31

**Authors:** Lili Guo, Bin Liu, Chencheng Chang, Fengying Ma, Le Zhou, Wenguang Zhang

**Affiliations:** 1 Inner Mongolia Engineering Research Center of Genomic Big Data for Agriculture, Inner Mongolia Agricultural University, Hohhot 010010, China; 13474912747@163.com (L.G.); changchencheng8112@163.com (C.C.); fengyingma1997@163.com (F.M.); zxcvbnm8880314@163.com (L.Z.); 2 Inner Mongolia Bionew Technology Co., Ltd., Hohhot 010010, China; liu207702@163.com; 3 College of Animal Science, Inner Mongolia Agricultural University, Hohhot 010010, China; 4 College of Life Science, Inner Mongolia Agricultural University, Hohhot 010010, China

**Keywords:** Bactrian camel, random forest, innate immune gene, gene co-expression network, RNA-seq

## Abstract

**Simple Summary:**

Bactrian camels survive extreme desert conditions—intense heat, scarce food, and little water—better than most animals. Scientists wanted to understand how their bodies defend against diseases across different organs like the liver, blood, and stomach. We studied genetic activity in 11 major body tissues and organs from over 100 camels. Using computer analysis, we identified unique genes that act like “ID cards” for each tissue—for example, fat tissue uses special genes to manage energy. Most importantly, we discovered that camels have different immune defense strategies in different body parts. Their liver uses one set of genes to filter germs, their blood uses another to fight infections quickly, and their stomach uses a third to handle gut bacteria. This “tailored defense system” helps camels stay healthy in the harsh desert. Understanding how camels naturally resist disease could help farmers raise healthier livestock in dry regions and may even inspire new ways to protect human health during environmental stresses like droughts or heatwaves.

**Abstract:**

The Bactrian camel exemplifies mammalian adaptation to deserts, but the spatial organization of its innate immune system remains uncharacterized. This study integrated transcriptomes from 110 samples across 11 major tissues and organs to resolve tissue-specific gene expression and innate immune modularity. Through differential expression analysis, Tau specificity index (τ > 0.8), and machine learning validation (Random Forest F1-score = 0.86 ± 0.11), we identified 4242 high-confidence tissue-specific genes (e.g., *LIPE*/*PLIN1* in adipose). Weighted gene co-expression network analysis (WGCNA) of 1522 innate immune genes revealed 11 co-expression modules, with six exhibiting significant tissue associations (FDR < 0.01): liver-specific (r = 0.96), spleen-adipose-enriched (r = 0.88), muscle-associated (r = 0.82), and blood-specific (r = 0.80) modules. These networks demonstrated multifunctional coordination of immune pathways—including Pattern Recognition, Cytokine Signaling, and Phagocytosis—rather than isolated functions. Our results establish that camel innate immunity is organized into spatially modular networks tailored to tissue microenvironments, providing the first systems-level framework for understanding immune resilience in desert-adapted mammals and may inform strategies for enhancing livestock resilience in arid regions.

## 1. Introduction

Bactrian camels (*Camelus bactrianus*) exemplify extraordinary mammalian adaptation to extreme desert environments, enduring temperature fluctuations, water scarcity, and nutritional stress [1,2]. Their evolution of unique physiological systems to maintain homeostasis under chronic stressors positions them as ideal models for studying molecular adaptation, particularly in immune regulation across heterogeneous tissue environments [3,4].

Innate immunity—the first line of defense against pathogens—is critical for tissue integrity and systemic homeostasis [5,6]. Emerging evidence reveals that innate immune regulation is not monolithic but exhibits spatial heterogeneity, shaped by tissue-specific microenvironments, mechanical stresses, metabolic demands, and localized microbial exposure [7,8,9,10]. This spatial variation suggests modular organization of immune networks, where functionally coherent gene sets (modules) operate in a tissue-contextualized manner [7,11]. However, the mechanisms by which such spatial modularity governs innate immunity in extreme environment-adapted mammal remains unexplored, especially in non-model organisms like camels.

Advances in transcriptomics and systems biology now enable the comprehensive mapping of gene expression landscapes across tissues. Integrating these data with machine learning and network-based analyses like weighted gene co-expression network analysis (WGCNA) [12] provides a powerful framework with which to uncover latent modular architectures, identify core regulatory genes, and delineate immune-functional units across tissues [13,14,15,16].

Tissues constitute fundamental units of biological structure and function, serving as critical sites for the initiation and regulation of immune responses. Due to differences in anatomical location, physiological function, and degree of environmental exposure, each tissue bears unique immunological responsibilities. For example, the spleen is a secondary lymphoid organ responsible for clearing pathogens [17], and the blood serves as a channel for the transport of immune cells [18]. The gastrointestinal tract and lungs are often exposed to high loads of microorganisms and antigens [19,20]. The liver plays a central role in immune tolerance and inflammation regulation [21]. Meanwhile, tissues like adipose, muscle, and kidney are increasingly recognized for their tissue-resident immune populations and specialized immunoregulatory functions [22,23,24].

In this study, we characterize the spatial modularity of innate immune networks across 11 major tissues and organs of Bactrian camels. We integrated multi-tissue transcriptomics with differential expression, tissue-specificity indices, machine learning, and WGCNA to: (1) identify tissue-specific gene signatures and functional markers defining tissue identity, (2) detect innate immune-related co-expression modules and their hub genes, and (3) examine the tissue-level associations of these modules to reveal spatial immune network organization.

Our work establishes a foundational framework for spatially resolved immune adaptation in desert mammals and examines how environmental stress may relate to modular immune responses across tissues.

## 2. Materials and Methods

### 2.1. Transcriptome Data Sources

This study analyzed a total of 110 samples from 11 distinct tissues and organs of Bactrian camels. Except for adipose tissue, all other samples were collected from discrete organs.

Whole blood samples (10 mL) were collected from the jugular vein of 10 adult domestic Bactrian camels in Alxa Left Banner, Inner Mongolia Autonomous Region, Bayanhot, China. Samples were preserved in TRIzol reagent (TaKaRa, Kusatsu, Shiga, Japan) at −80 °C prior to RNA extraction. Total RNA (1.0 μg per sample) was used to construct sequencing libraries with the TruSeq RNA Library Prep Kit v2 (Illumina, San Diego, CA, USA). Polyadenylated mRNA was enriched by poly-T magnetic beads, fragmented, and converted to double-stranded cDNA. Libraries underwent end repair, A-tailing, adapter ligation, and PCR amplification. Quality was verified using an Agilent Bioanalyzer (Agilent Technologies, Santa Clara, CA, USA), followed by normalization and pooling.

Sequencing was performed on the Illumina NovaSeq 6000 platform (150 bp paired-end reads) using the TruSeq PE Cluster Kit v3-cBot-HS (Illumina, San Diego, CA, USA).

Transcriptomic datasets from 100 additional camel tissue samples were retrieved from NCBI SRA (Projects: PRJNA471391, PRJNA857334, PRJNA485657, PRJNA416060). Tissues included: adipose (28), stomach (12 chambers 1, 5 chambers 2, 2 chambers), intestine (6 small intestine, 2 cecum), liver (8), kidney (7), spleen (7), skeletal muscle (7), myocardium (6), esophagus (5), and lung (5) (Table S1).

### 2.2. Transcriptome Data Processing

Raw reads were assessed using FastQC (v0.11.9) for base quality, GC content, and duplication levels. Adapters and low-quality reads (Q20 < 80% or N bases > 10%) were trimmed using Fastp [25] (v0.23.2).

Clean reads were aligned to the *C. bactrianus* reference genome (Ca_bactrianus_MBC_1.0) via HISAT2 [26,27] (v2.0.5) with default parameters. Alignments were sorted and converted to BAM format using Samtools [28] (v1.12). Transcript assembly and expression quantification (raw counts, TPM) were performed with StringTie [27] (v1.3.3). Gene expression levels were quantified using both raw gene counts and TPM (Transcripts Per Mil-lion), allowing for flexible downstream analysis.

### 2.3. Identification of Tissue-Specific Differentially Expressed Genes

Based on RNA-seq data from 110 samples across different camel tissues, we identified tissue-specific gene expression profiles. To enhance the efficiency and reliability of differential analysis, lowly expressed genes (expression count < 10 in more than 80% of samples) were filtered out.

Batch effects were corrected using the ComBat algorithm, and differential expression analysis was conducted using DESeq2 (v1.38.3). Pairwise comparisons were performed between each tissue and all other tissues to identify uniquely expressed genes. Genes were considered differentially expressed if they met the following criteria: absolute log_2_ fold change (|log_2_FC|) > 1 and an adjusted *p*-value (*P*_adj_) < 0.05, corrected using the Benjamini–Hochberg method.

We also calculated the Tau index, which quantifies the tissue specificity of gene expression. Tau values range from 0 (ubiquitous expression) to 1 (exclusive expression in a single tissue). Genes with Tau > 0.8 were considered highly tissue-specific.

The Tau index was calculated as follows:

(1) $$\tau =\frac{\sum_{i=1}^{n}\left(1-\frac{x_{i}}{max\left(x\right)}\right)}{n-1}$$

where

$x_{i}$ is the expression of a gene in tissue,

$max\left(x\right)$ is the maximum expression across all tissues,

n is the total number of tissues.

### 2.4. Machine Learning-Based Identification of Tissue-Specific Genes

To refine the set of tissue-specific genes, we applied three machine learning models: Random Forest (RF), Extreme Gradient Boosting (XGBoost), and Support Vector Machine (SVM), using the gene set identified by both DESeq2 and the Tau index.

The dataset (110 samples) was partitioned into a training set (70%, n = 77) and a held-out test set (30%, n = 33). To address class imbalance in the training set, Synthetic Minority Over-sampling Technique (SMOTE) [29] was applied. Hyperparameter optimization was performed via 5-fold cross-validation on the training set. Final model performance was assessed on the independent test set using macro-averaged precision, recall, and F1-score. Confusion matrices comparing predicted versus true tissue labels.

All machine learning analyses were conducted in Python (v3.10). Data processing was done using Pandas (v1.5.3); model training and evaluation were performed using Scikit-learn (v1.2.2); visualizations were generated with Matplotlib (v3.7.1) and Seaborn (v0.12.2).

### 2.5. Construction of Innate Immune Gene Co-Expression Networks

Weighted Gene Co-expression Network Analysis (WGCNA) was employed to construct tissue-level co-expression networks for 1522 innate immune-related genes across 11 tissues [12]. The identification process of the 1522 innate immune genes is described in detail in an unpublished article [30]. Pairwise Pearson correlation coefficients were computed for all gene pairs, followed by transformation into a signed adjacency matrix using a soft-thresholding power β = 12. This threshold was selected to achieve scale-free topology fit (R^2^ > 0.85). The adjacency matrix was subsequently converted to a Topological Overlap Matrix (TOM), which quantifies gene-pair similarity through both direct correlations and shared neighborhood connectivity. Hierarchical clustering with average linkage was performed on the TOM-based dissimilarity matrix (1-TOM), and co-expression modules were identified via dynamic hybrid tree cutting with a minimum module size of 20 genes. All analyses were implemented in R (v4.2.2) using the WGCNA package (v1.72.5). Resulting networks were exported to Cytoscape (v3.10.2) for visualization.

### 2.6. Gene Enrichment Analysis

Functional enrichment analysis of selected gene sets was conducted using g:Profiler [31] (https://biit.cs.ut.ee/gprofiler/gost (accessed on 25 March 2025)). This web-based platform integrates various biological databases, including Gene Ontology (GO), Kyoto Encyclopedia of Genes and Genomes (KEGG), Reactome, and transcription factor binding sites.

Statistical significance was determined by Benjamini–Hochberg false discovery rate (FDR) correction, with *P*_adj_ < 0.05 considered significant. Only terms containing ≥5 annotated genes from the input set were analyzed.

## 3. Results

### 3.1. Overview of Transcriptome Data

A total of 100 transcriptome datasets from various Bactrian camel tissues were obtained from the NCBI Sequence Read Archive (SRA), along with 10 additional whole-blood transcriptomes generated in this study. In total, approximately 645 GB of raw sequencing data were collected. After quality control, the effective base rate reached 97.64%, with a Q30 ratio above 92.72% and a GC content exceeding 51.74%. On average, 91.85% of reads were aligned to the Bactrian camel reference genome (Ca_bactrianus_MBC_1.0), and about 80.26% of reads per sample were uniquely mapped. Following read alignment and quantification, genes not expressed in at least 80% of samples and with gene counts < 10 were filtered out, resulting in 20,957 genes being retained for downstream analysis.

### 3.2. Tissue-Specific Gene Expression

To systematically identify tissue-specific genes, we employed an integrated approach combining differential expression analysis, Tau index filtering, and machine learning-based classification.

**Figure 1:**
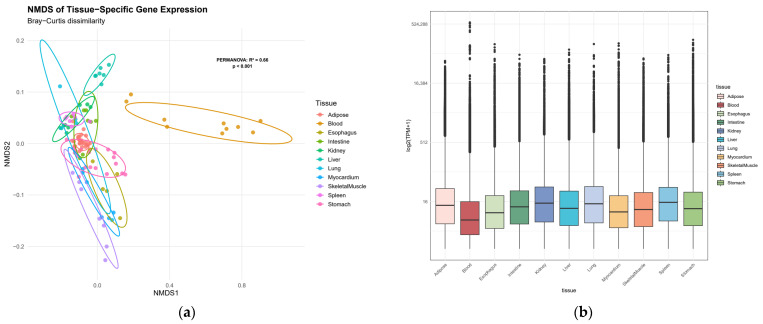
Transcriptomic landscape across Bactrian camel tissues. (**a**) Non-metric multidimensional scaling (NMDS) plot of tissue-specific gene expression profiles, points represent individual samples, colored by tissue type; (**b**) distribution of tissue-specific expression levels by box plot.

First, the tissue distribution of TPM values for all 20,957 genes was examined. A global heterogeneity test using PERMANOVA revealed that tissue type significantly influenced gene expression profiles (R^2^ = 0.66, F = 19.2, *p* < 0.001), indicating substantial transcriptomic divergence across tissues (Figure 1).

Differential gene expression analysis was performed using DESeq2 for each of the 11 tissues. The number of tissue-specific DEGs identified were as follows: skeletal muscle (3343), stomach (2737), spleen (1207), kidney (923), lung (666), liver (653), esophagus (513), intestine (483), adipose tissue (1587), myocardium (372), and blood (162), totaling 12,646 differentially expressed genes (some redundantly counted across tissues).

Tau index analysis showed that approximately 77.2% of genes had Tau values between 0.5 and 1.0, indicating widespread tissue-specific expression bias. Using stringent criteria (Tau > 0.8 and con-firmed by DE analysis), a set of 4242 high-confidence tissue-specific genes was identified (Table S2).

**Figure 2:**
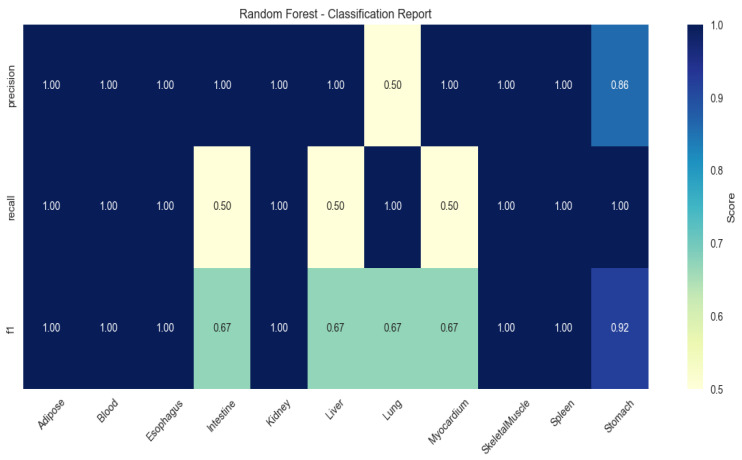
Heatmap of tissue classification performance using Random Forest algorithm.

To validate the discriminative power of these genes, multi-class classification models were trained using Random Forest (RF), Extreme Gradient Boosting (XGBoost), and Support Vector Machine (SVM). RF achieved peak performance (mean F1-score: 0.86 ± 0.11), with perfect classification (F1 = 1.00) for adipose, kidney, and spleen (Figure 2).

**Figure 3:**
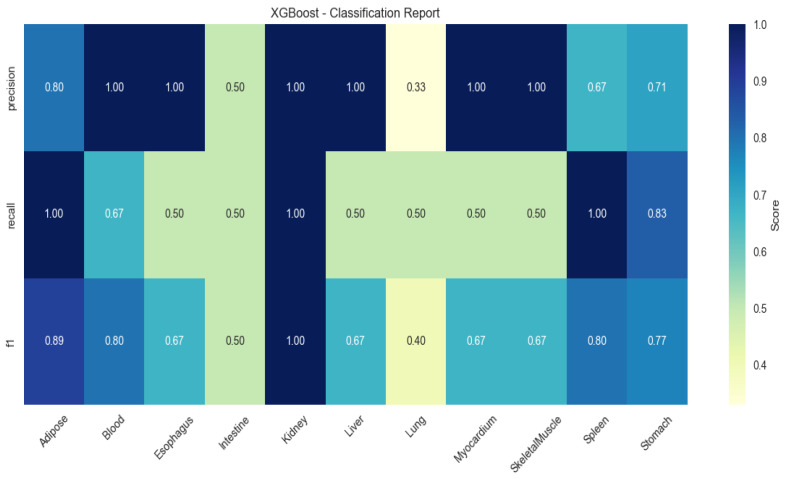
Heatmap of tissue classification performance using XGBoost algorithm.

XGBoost showed elevated performance in lung (F1 = 0.83) and myocardium (F1 = 0.67), but higher variability (SD ± 0.21; Figure 3).

**Figure 4:**
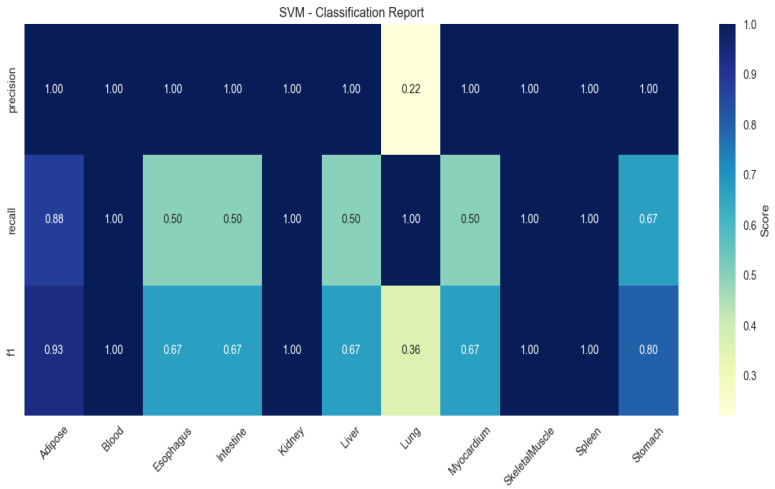
Heatmap of tissue classification performance using Support Vector Machine (SVM) algorithm.

SVM demonstrated limited generalizability (mean F1 = 0.67), particularly in blood (recall = 0.50) and myocardium (recall = 0.60; Figure 4).

**Figure 5:**
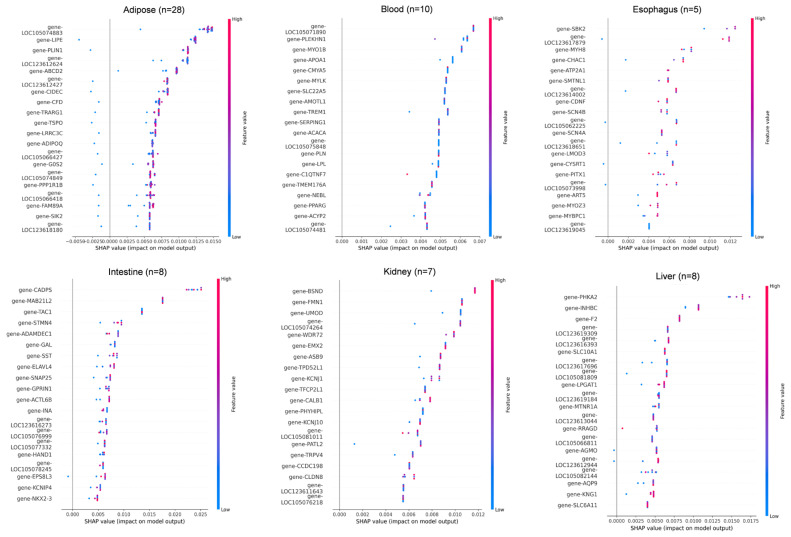

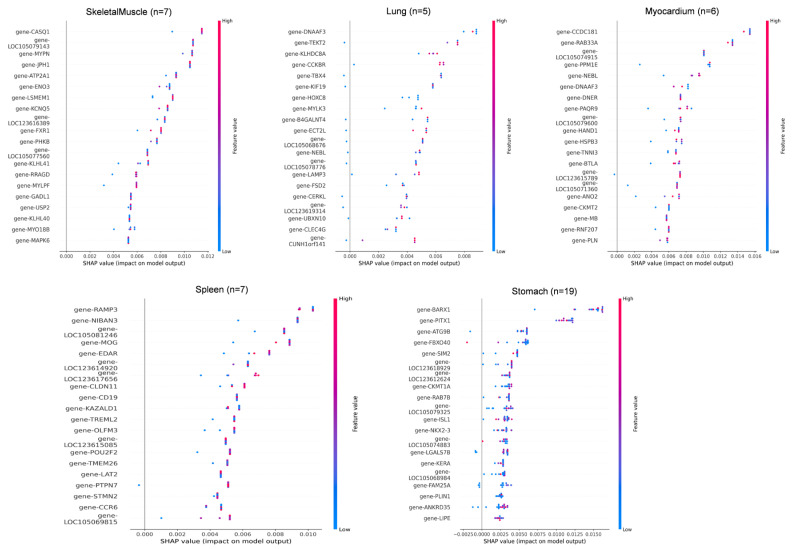
SHAP value distribution of top discriminative genes across 11 tissues. SHAP value summary plot depicting the impact of each of the most important genes on model output. Gene expression (shown by coloring) as a function of SHAP value (x-axis) showing the relation between expression pattern and model’s prediction.

To further interpret the model, SHapley Additive exPlanations (SHAP) were used to analyze feature importance across the 11 tissue types. The top 20 most influential genes contributing to tissue classification are shown in Figure 5 and Table S3.

In adipose tissue (n = 28), *LOC105074883*, *LIPE*, and *PLIN1* showed the highest SHAP contributions, consistent with their roles in lipid metabolism. In blood (n = 10), key genes included *LOC105071890*, *PLEKHN1*, and *MYO1B*, all showing SHAP values in the 0.004–0.007 range. In esophagus (n = 5), *SBK2*, *LOC123617879*, and *MYH8* were the dominant predictors, though sample scarcity led to greater SHAP dispersion. For intestine (n = 8), *CADPS*, *MAB2112*, and *TAC1* showed strong positive impacts on classification. In kidney (n = 7), *BSND*, *FMN1*, and *UMOD* were top contributors, reflecting roles in ion transport and metabolism. In liver (n = 8), core genes included *PHKA2*, *INHBC*, and *F2*, with *SLC10A1* showing a linear in-crease in SHAP value relative to expression. In lung (n = 5), *DNAAF3*, *TEKT2*, and *KLHDC8A* had narrow SHAP ranges, and misclassification with myocardium was observed, possibly due to functional overlap. Additional key drivers included *CCDC181* and *RAB33A* in myocardium, *CASQ1*, *MYPN*, and *LOC105079143* in skeletal muscle, *RAMP3* and *NIBAN3* in spleen, and *BARX1*, *PITX1*, and *ATG9B* in stomach.

### 3.3. Co-Expression Network of Innate Immunity Genes Across Tissues

To explore tissue-specific co-expression of innate immune genes, we performed Weighted Gene Co-expression Network Analysis (WGCNA) on the 1522 expressed innate immune genes across 110 samples. Using a soft-thresholding power of β = 14, a scale-free topology was achieved (R^2^ = 0.85). This analysis identified eleven distinct co-expression modules, with sizes ranging from 31 to 335 genes.

**Figure 6:**
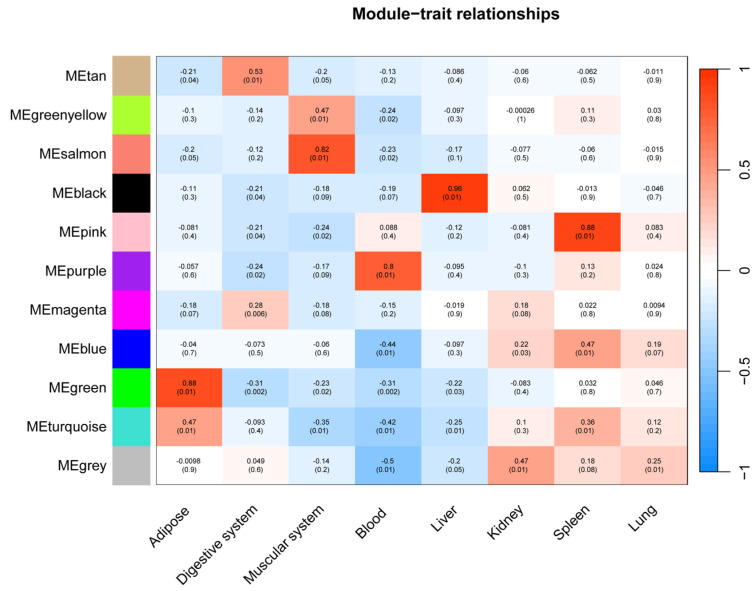
Correlation between innate immunity gene expression clusters and tissue types. Each row represents a module, with the color key on the left denoting module identity. The heatmap depicts the correlation coefficient between each module eigengene and tissue type, ranging from negative (blue) to positive (red). Correlation *p*-values are indicated within each cell.

Among these, six modules exhibited significant correlations with specific tissues (*p* < 0.01) (Figure 6), and functional enrichment analysis revealed distinct biological processes underpinning their tissue-specific immune functions.

**Figure 7:**
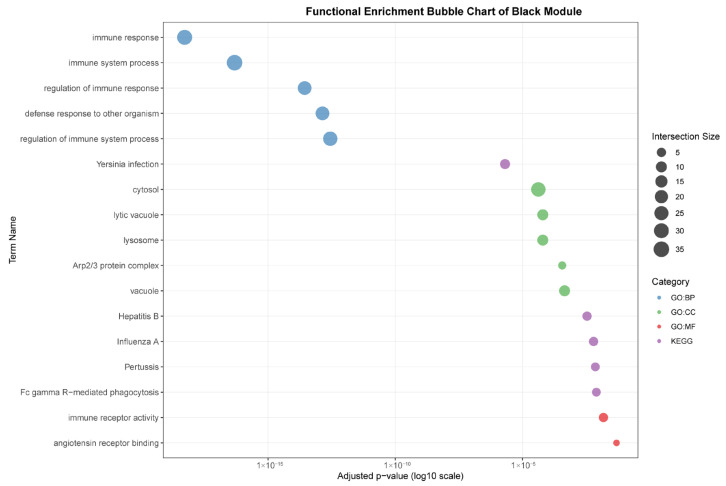

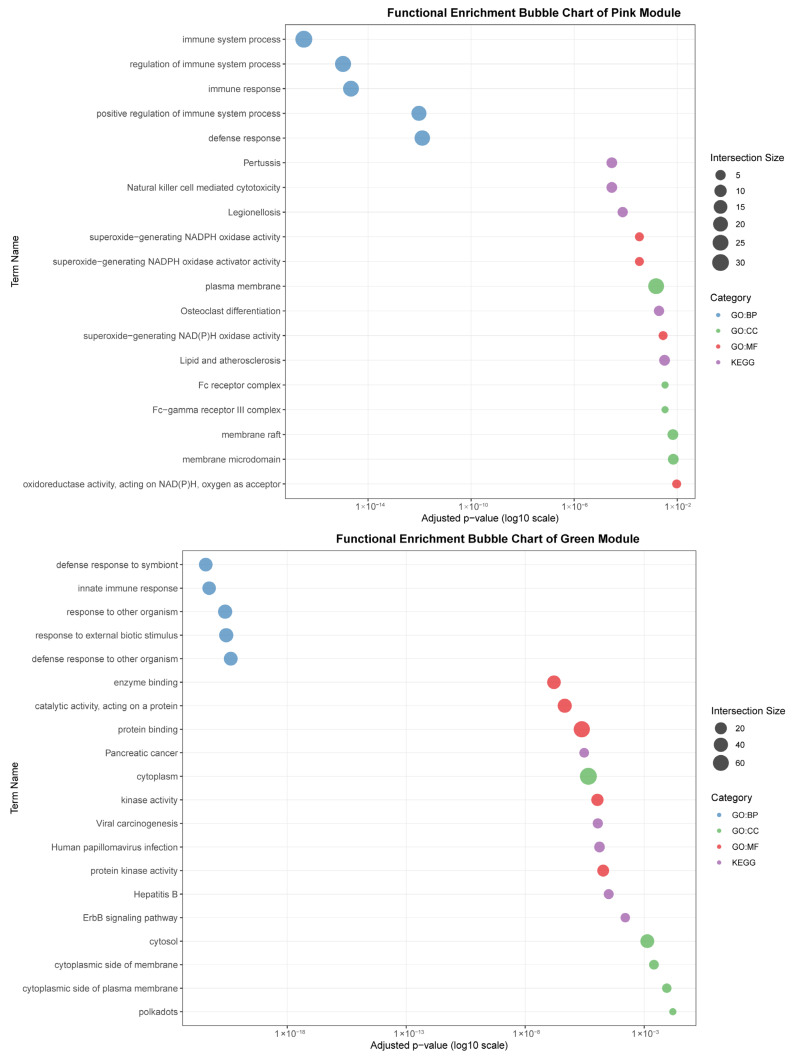

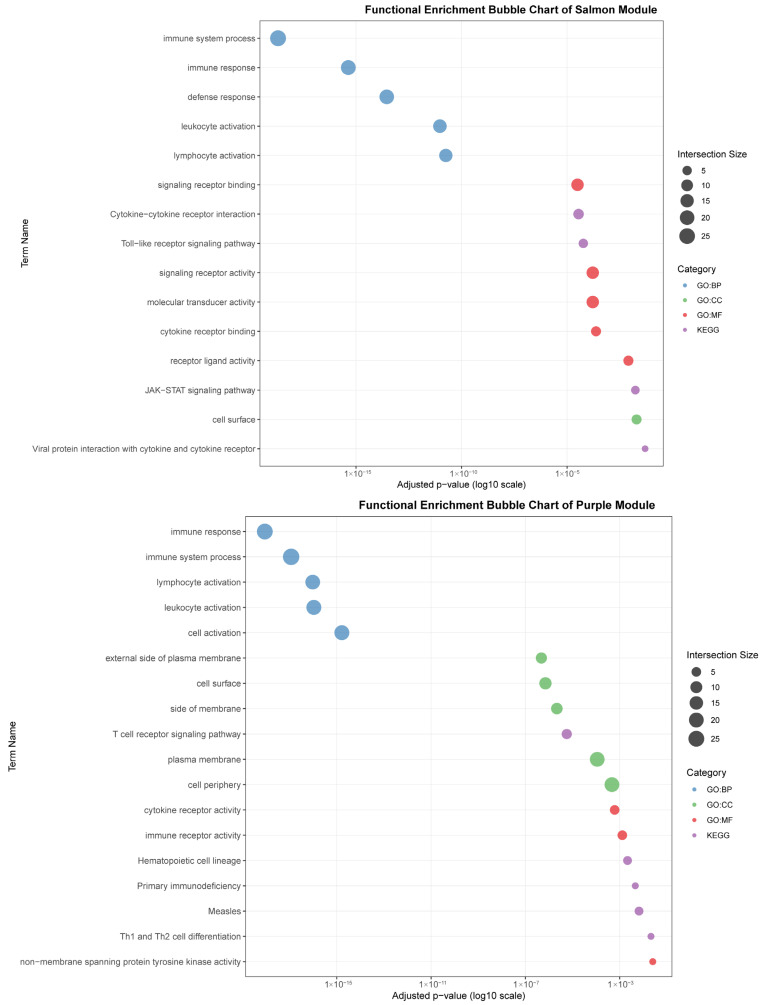

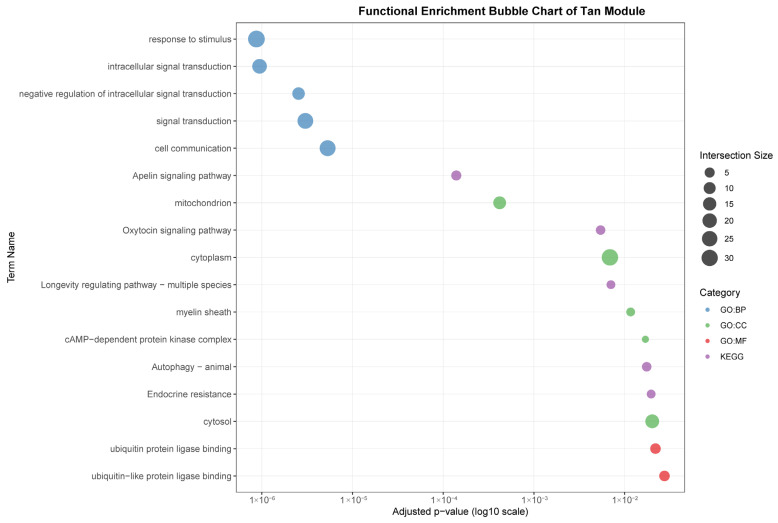
Functional enrichment analyses on six tissue-associated modules. Color represents the term category. The size of the dots represents the number of genes.

As seen in Figure 7, the black module (88 genes), highly correlated with liver (r = 0.96), was predominantly enriched for core immune functions, including “immune response,” “defense response to other organism,” and pathways related to specific infections such as “Yersinia infection” and “Hepatitis B.” The association with cellular components like “lysosome” and “lytic vacuole” underscores this module’s role in hepatic pathogen clearance and immune activation.

The pink module (282 genes) showed a strong correlation with the spleen (r = 0.88). It was characterized by terms related to broad immune regulation (“immune system process,” “defense response”) and effector functions, such as “Fc receptor complex” and “superoxide-generating NADPH oxidase activity.” Its enrichment in pathways like “Natural killer cell mediated cytotoxicity” and “Osteoclast differentiation” highlights its role in coordinating innate and adaptive immune responses within the spleen.

The green module (95 genes) was strongly associated with adipose tissue (r = 0.88). Its functional profile suggests an interface between metabolic signaling and immune defense, being enriched for “innate immune response,” “defense response to symbiont,” and specific kinase activities. Pathways like “ErbB signaling pathway” and “Viral carcinogenesis” further indicate a unique role for adipose tissue in integrating metabolic and anti-viral responses.

The salmon module (31 genes) was correlated with muscle (r = 0.82) and was notably enriched for signaling pathways, including “Cytokine-cytokine receptor interaction,” “Toll-like receptor signaling pathway,” and “JAK-STAT signaling pathway.” This points to a significant, albeit less explored, role for muscle in immune signaling and systemic inflammation modulation.

The purple module (44 genes), linked to blood (r = 0.80), was defined by lymphocyte and cell surface activities. Key terms included “lymphocyte activation,” “T cell receptor signaling pathway,” and “cell surface.” Its connection to KEGG pathways such as “Primary immunodeficiency” and “Th1 and Th2 cell differentiation” emphasizes its critical function in blood-cell-mediated immunity and T-cell communication.

The tan module (35 genes) demonstrated a weaker but significant correlation with gastrointestinal (r = 0.53). In contrast to the immune-centric modules, it was enriched for intracellular signaling and metabolic processes, such as “intracellular signal transduction,” “Apelin signaling pathway,” and cellular components like “mitochondrion.” This suggests a primary role in metabolic regulation and local signal modulation within the GI tract, with indirect links to immune homeostasis.

In summary, our integrated analysis demonstrates that innate immune genes are organized into cohesive co-expression modules that exhibit strong tissue specificity. The distinct functional enrichment of each module aligns with the physiological and immunological roles of their corresponding tissues, revealing a sophisticated, modular architecture of the innate immune system across the body.

### 3.4. Tissue-Specific Innate Immunity Networks

**Figure 8:**
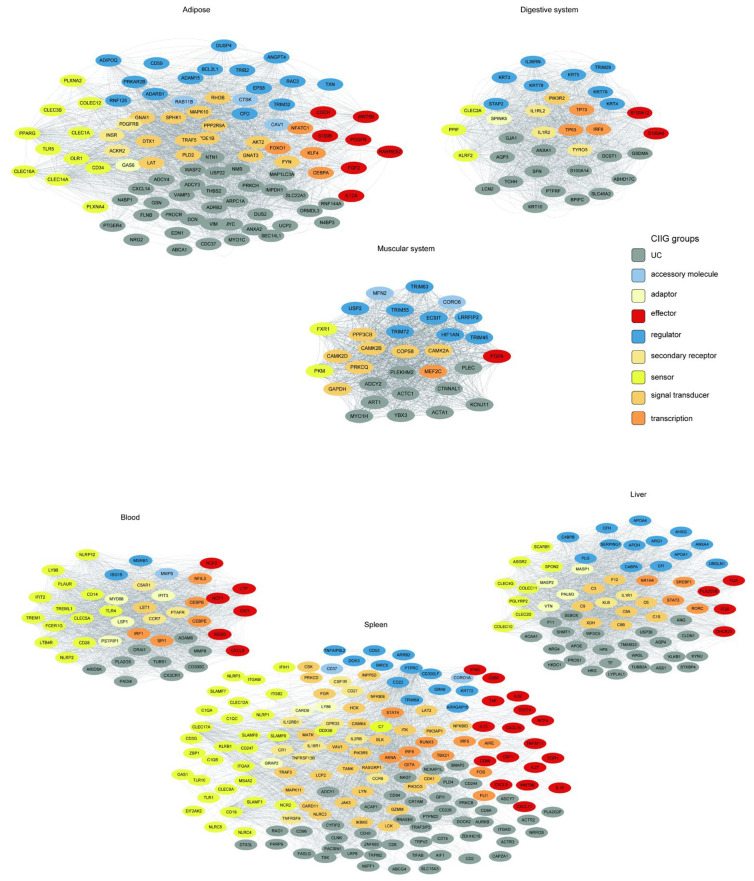
Tissue-resolved innate immunity networks. Co-expression networks for six tissue-associated modules. Nodes represent innate immunity genes colored by functional class (see legend). Edge thickness scales with topological overlap (TOM).

To elucidate the functional coordination within tissue-associated innate immunity modules, we constructed and visualized tissue-resolved co-expression networks for the six significant WGCNA modules identified in our analysis (Figure 8). Crucially, each module corresponds directly to a specific physiological system or organ, as defined by our initial tissue sampling and module-trait relationship analysis (see Section 2). The networks were derived from topological overlap matrices (TOM), with tissue-specific edge thresholds applied to filter out background noise and ensure that the resulting networks reflect biologically relevant, high-confidence interactions.

The tissue-specific innate immunity networks were defined by their corresponding WGCNA modules, where the green, tan, salmon, purple, black, and pink modules represent the innate immunity co-expression networks for adipose tissue, digestive system, muscular system, the blood, the liver, and the spleen, respectively.

We employed a tiered stringency approach for network construction. For the adipose (24 genes), digestive system (35 genes), muscular system (31 genes), and blood (44 genes) modules, we utilized average TOM thresholds, yielding coherent networks with 2106, 1842, 1570, and 1955 significant edges, respectively. In contrast, the liver (88 genes) and spleen (282 genes) modules, being substantially larger and more complex, required higher stringency thresholds (TOM > 0.06 and >0.18, respectively) to distill the most robust interactions, resulting in focused yet dense networks of 1964 and 2134 edges.

We found that innate immune genes from distinct functional categories (color-coded by function in Figure 8) formed unique connectivity patterns in each tissue. This resulted in tissue-specific “interactomes,” in which genes involved in pathogen perception, signaling, and effector functions were connected in distinct topological patterns. This rewiring likely underlies the specialization of the innate immune response, enabling the gastrointestinal system to manage commensal and pathogenic microbes differently from how the liver handles systemic inflammation or the adipose tissue integrates immunometabolic signals.

## 4. Discussion

This study integrates multi-tissue transcriptomic data with machine learning to systematically delineate the expression dynamics and regulatory networks of tissue-specific and innate immune genes in the Bactrian camel. By integrating differential expression analysis with machine learning and network biology, we uncover two fundamental principles of camel biology: first, the existence of a robust repertoire of tissue-specific genes that define organ identity and functional specialization; and second, a striking spatial modularity in the innate immune system, where coordinated gene networks are tailored to the unique microenvironment of each tissue. These findings provide a novel systems-level framework for understanding how physiological specialization and immune function are integrated in a mammal adapted to extreme environments.

Our integrated approach, combining DESeq2-based differential expression analysis with Tau index filtering, identified 4242 high-confidence tissue-specific genes. The functional roles of the identified tissue-specific genes powerfully reflect the physiological priorities of each tissue within the camel’s extreme niche. In adipose tissue, the co-expression of *LIPE*, *PLIN1* (as known to be involved in lipolysis and fatty acid oxidation of dairy cows [32]), and *ADIPOQ* (current human and animal studies have reported that *ADIPOQ* is anti-inflammatory factor [33]) suggests a finely tuned program that not only mobilizes energy reserves but also actively constrains systemic inflammation—a crucial adaptation in an environment where metabolic and immune resources must be judiciously allocated. Similarly, the high SHAP contribution of *MYO1B* (myosin IB) and *PLEKHN1* in blood underscores their importance as effector genes in lymphocyte polarization and neutrophil extracellular trap (NET) formation [34,35], respectively, highlighting their utility as biomarkers for dynamic immune cell states.

Critically, the Random Forest classifier, trained on these tissue-specific gene expression profiles, achieved exceptional performance (mean F1-score = 0.86). This not only validates the reliability of the identified gene set but also strongly supports the concept that transcriptomic profiles serve as robust biological signatures of tissue identity and functional state. SHAP value analysis further elucidated the key genetic drivers of tissue classification, offering mechanistic insights into the molecular basis of tissue-specific functions.

Weighted Gene Co-expression Network Analysis (WGCNA) of 1522 expressed innate immune genes across 110 samples identified 11 co-expression modules, six of which exhibited significant correlations with specific tissues (*p* < 0.01). This striking pattern demonstrates a pronounced spatial modularity in innate immune regulation, where transcriptional programs are tailored to the unique microenvironmental pressures, pathogen exposures, and metabolic demands encountered by each tissue [36].

The liver’s dominant correlation with the black module aligns with its dual role as a central metabolic/detoxification organ and a key immunological site. This module likely encodes pathways for hepatic immune tolerance induction and inflammation modulation in response to exogenous and endogenous stressors [37]. As a secondary lymphoid organ, the spleen’s strong association with the pink module reflects finely tuned gene expression programs essential for pathogen clearance and immunological memory formation [17]. Correlation with the green module supports adipose tissue’s endocrine function in systemic immune regulation, potentially mediated through adipokine and cytokine signaling [38]. The muscle-associated salmon module potentially governs immune functions critical for tissue repair, inflammation control, and metabolic adaptation within musculature [39]. The blood-specific purple module likely mirrors the functional status and compositional heterogeneity of circulating immune cells [18]. The weaker but significant correlation with the tan module suggests the existence of a dedicated immune network within the GI tract, presumably adapted to manage the complex luminal microbiota and antigenic load.

The architecture of the tissue-specific co-expression networks themselves reveals a higher-order organizational principle: innate immunity is executed not by isolated linear pathways, but by deeply integrated, multifunctional networks [40,41,42]. In every tissue-associated module we observed a tight interplay between genes for Pattern Recognition, Cytokine Signaling, and Immunoregulation. This design, evident in our networks from the GI tract to the blood, suggests that each tissue possesses a pre-wired, integrated response system. Rather than assembling a response from scratch, a tissue can deploy a coordinated program fine-tuned by evolution to its most common challenges [43,44,45]. We propose that this “plug-and-play” network strategy is a fundamental adaptation for resilience, enabling a rapid and appropriately scaled reaction to local stimuli without necessitating a costly, body-wide inflammatory state. For a camel in a resource-poor desert environment, the energetic and homeostatic cost of uncontained inflammation could be fatal.

Several limitations of this study highlight exciting avenues for future research. First, First, although intestinal segments consistently form distinct clusters distinct from other tissues—a pattern observed in the human GTEx project [46] and in multi-tissue transcriptome maps of cattle [47] and sheep [48]—the roles of nutrient processing and immune regulation in different intestinal regions require further investigation. Second, although tissue-specific transcriptional profiles remain stable postmortem and inter-tissue variability is greater than inter-individual variability [46], future studies should incorporate the effects of sex and age on disease-associated genes into more comprehensive models. Finally, while our network analysis provides a strong correlative foundation, the precise regulatory logic and causal relationships within these modules await functional validation through techniques such as single-cell RNA sequencing, CRISPR-based perturbations, and in vitro challenge experiments in tissue-specific contexts.

Collectively, these findings establish a crucial foundational framework for understanding the molecular basis of the Bactrian camel’s extraordinary resilience. The identified gene signatures, characterized co-expression modules, and resolved tissue-specific networks provide essential resources for future investigations. These include functional validation of key genes, mechanistic studies of tissue-specific immune adaptations and exploration of the genetic underpinnings of this species’ unique environmental adaptations.

## 5. Conclusions

Multi-tissue transcriptomics revealed spatially organized innate immunity in Bactrian camels. We identified 4242 tissue-specific genes validated by machine learning and discovered six functionally distinct immune modules (via WGCNA) strongly associated with liver, spleen, blood, muscular system, adipose tissues, and digestive system. These modules demonstrate cross-pathway integration as a core mechanism maintaining tissue immune homeostasis. This pioneering construction of multi-tissue co-expression networks decodes the spatial immune adaptation underlying camel resilience to extreme environments.

## Data Availability

These RNA-seq data are deposited in the NCBI Sequence Read Archive (SRA) under bio project number PRJNA1127254, PRJNA471391, PRJNA857334, PRJNA485657, PRJNA416060.

## Supplementary Materials

The following supporting information can be downloaded at: https://www.mdpi.com/article/10.3390/ani15213173/s1, Table S1: Summary of transcriptome dataset characteristics. Table S2: 4242 high-confidence tissue-specific genes. Table S3: Top five tissue-specific marker genes per tissue identified by SHAP analysis.
